# African Swine Fever Re-Emerging in Estonia: The Role of Seropositive Wild Boar from an Epidemiological Perspective

**DOI:** 10.3390/v13112121

**Published:** 2021-10-21

**Authors:** Katja Schulz, Jana Schulz, Christoph Staubach, Sandra Blome, Imbi Nurmoja, Franz J. Conraths, Carola Sauter-Louis, Arvo Viltrop

**Affiliations:** 1 Institute of Epidemiology, Friedrich-Loeffler-Institut, Federal Research Institute for Animal Health, Südufer 10, 17498 Greifswald-Insel Riems, Germany; Jana.Schulz@fli.de (J.S.); Christoph.Staubach@fli.de (C.S.); Franz.Conraths@fli.de (F.J.C.); Carola.Sauter-Louis@fli.de (C.S.-L.); 2 Institute of Diagnostic Virology, Friedrich-Loeffler-Institut, Federal Research Institute for Animal Health, Südufer 10, 17498 Greifswald-Insel Riems, Germany; Sandra.Blome@fli.de; 3 Estonian Veterinary and Food Laboratory (VFL), Kreutzwaldi 30, 51006 Tartu, Estonia; Imbi.Nurmoja@vetlab.ee; 4 Institute of Veterinary Medicine and Animal Sciences, Estonian University of Life Science, Kreutzwaldi 62, 51014 Tartu, Estonia; Arvo.Viltrop@emu.ee

**Keywords:** African swine fever, Estonia, ASFV-carrier, confidence in freedom, detection probability, sensitivity of surveillance, wild boar, cluster analysis

## Abstract

African swine fever (ASF) emerged in Estonia in 2014. From February 2019 to August 2020, no pigs or wild boar tested positive for ASF virus (ASFV), only ASFV-specific antibodies could be detected in shot wild boar. However, ASF recently re-emerged in wild boar. We tested three hypotheses that might explain the current situation: (i) ASFV may have been present throughout, but at a prevalence below the detection limit; (ii) seropositive wild boar may have remained infectious (i.e., virus-carriers) and kept the epidemic going; or (iii) ASF was gone for 1.5 years, but was recently re-introduced. Using Estonian surveillance data, the sensitivity of the surveillance system and the confidence in freedom from ASF were estimated. Furthermore, the detection probability was determined and cluster analyses were performed to investigate the role of serological positive wild boar. The results suggest that the surveillance system was not able to detect virus circulation at a design prevalence below 1%. With respect to the confidence in freedom from ASF, the results indicate that circulating virus should have been detected over time, if the prevalence was ≥2%. However, the decreasing wild boar population density and ongoing surveillance activities made ASFV circulation at a low prevalence unlikely. Cluster analyses provided no evidence for a significant accumulation of serologically positive wild boar in temporal connection to the re-emergence of ASFV. Further targeted research, such as long-term experimental studies and molecular epidemiology, is necessary to improve our knowledge on the epidemiology of ASF and to control the disease more effectively.

## 1. Introduction

African swine fever (ASF) was first described by Montgomery [1] in 1921. In the following decades, ASF virus (ASFV) mainly circulated in Africa. However, it was introduced into Europe on various occasions since 1957, but had almost completely been eliminated from the European continent by 1995 [2,3]. It was only in Sardinia that ASF became endemic; it has been constantly present on the island since 1978 [4]. In 2007, ASFV of genotype II was introduced into Georgia, where it spread over various European countries, both inside and outside of the European Union (EU); it reached China and several other Asian countries in recent years [5]. After ASF appeared in Lithuania, Poland, and Latvia in the first half of 2014, Estonia has been affected since September 2014 [6,7,8,9].

In contrast to previous outbreak situations in Europe, where mostly domestic pigs were affected with some spillover infections into wild boar, ASF mainly circulates among wild boar in the current epidemic in Europe, described as the “Wild Boar–Habitat Epidemiologic Cycle” [10]. Controlling ASF in wild boar is incomparably more difficult than in domestic pigs, which usually constitute a defined population in a distinct area, normally the premises of a farm. Yet, controlling ASF is much more challenging in wild boar, and the surveillance of the disease is more complex in these animals. The case/fatality ratio of ASF is extremely high, which leads to a significantly higher detection probability in wild boar found dead [7,11,12]. Thus, surveillance activities focus on passive surveillance, i.e., the detection of wild boar carcasses and testing them for ASF. However, the probability of detecting wild boar carcasses depends on a variety of factors, including land-use, coverage of the area with crops, grassland, shrubs, bushes, or forest, and the decomposition speed of the carcasses as a function of temperature, the presence of scavengers, insects, etc. Although the number of dead wild boar is expected to increase during an ASF epidemic, the animals often hide when they feel sick, and die; thus, making discovery more difficult. Moreover, carcasses may be hidden or eaten by scavengers, also reducing the potential for discovery [13,14]. This makes successful passive surveillance labor-intensive. Furthermore, in countries such as Estonia, where ASF has been present in wild boar for several years throughout most of the country, and since around 50% of the country is forest and wetland, maintaining surveillance at a high level is a major challenge. By combining reduced intensive surveillance efforts with a decreased population density, as a result of the high lethality due to ASFV infection [15], it is obvious that the probability of detecting wild boar infected with ASF decreases in such a situation. Yet, passive surveillance is usually supplemented with active surveillance, i.e., the sampling and testing of wild boar that were apparently healthy when shot. In an advanced stage of an ASF epidemic, it was found that the number of hunted wild boar that tested positive for ASFV-specific antibodies (i.e., seropositive wild boar) increased, and were, at the same time, negative for ASFV. This was probably due to an accumulation of the number of animals that had survived the infection [16,17]. Thus, active surveillance is an essential addition to passive surveillance, especially in a later phase of an ASF epidemic.

In Estonia, the last ASF outbreaks in domestic pig holdings for several years occurred in September 2017 [18]. However, in July 2021, one domestic pig farm had to report an ASF outbreak and, thus, the long period without ASF in domestic pigs ended. By contrast, wild boar that tested ASFV-positive, seropositive, or both, were regularly detected until February 2019. Thereafter, only seropositive, but not ASFV-positive wild boar, were found for more than 1 year, suggesting a lack of new ASFV infections and, thus, a potentially subsiding epidemic [17]. In August 2020, however, an ASFV-positive wild boar was found in Rapla County in the central part of Estonia, followed by several detections of ASFV-positive wild boar in this region. Shortly afterwards, new ASFV cases were detected in Lääne-Viru County in the northeast of the country, around 120 km from the cases in Rapla County. The re-emergence of ASFV-positive wild boar after a time, when there was reason to hope that the elimination of ASF from Estonia might be possible, fueled controversial discussions regarding the detection probability of circulating ASFV, and the role of seropositive wild boar in the ASF epidemic. Before discussing the latter, agreement must be reached on the definition of so-called ASFV-carriers. According to Putt, et al. [19], a true virus-carrier is an infected individual that sheds the pathogen, but neither sickens nor shows any clinical signs. There is general consent in the scientific community—that after infection with ASFV, infected individuals shed the pathogen from 24–48 h post infection, until at least 35 days. During that time, laboratory-testing yields ASFV-positive test results. In some laboratory experiments, ASFV was (intermittent) detectable up to 100 days post-infection [20,21]. After about 7 to 10 days, antibodies are formed and, thus, there is a time period, in which ASFV- and serologically-positive laboratory test results are observed simultaneously [5,22]. In case of survival of the infected wild boar, it will clear the infection and laboratory testing usually yields seropositive, but ASFV-negative test results. Petrov et al. [20] and Nurmoja, et al. [23] showed that, in their experiments with genotype I and II strains, ASF survivors did not transmit the virus to sentinels commingled later than 50 days post-infection. After 100 days of survival, no virus was found in the respective animals, but genome copies could be detected. Some scientists would define wild boar that shed ASFV up to 100 days post-infection as an ASFV-carrier. However, another group of experts discussed whether ASFV could be reactivated in a serological positive, but ASFV-negative wild boar, at a later point in time, e.g., in case of immunosuppression, stress, or death. Thus, there is a debate whether serologically-positive wild boar should be considered as potential ASFV-carriers that might play a role in maintaining the epidemic. Blome et al. [5] doubt that genome copies have a significant impact on disease spread and transmission dynamics. These findings and the resulting different definitions of a (potential) ASFV-carrier emphasize once again the importance of a similar understanding of terminology. Despite the controversial discussion, there is currently no evidence that wild boar that have survived an ASF infection play a major long-term role in the maintenance of ASF [24]. The same is probably true for domestic pigs under field conditions. Very recently, Oh et al. [25] conducted a long-term follow-up of convalescent pigs and their offspring in Vietnam, and did not observe any transmission events. 

This scientific discussion and the course of the epidemic in Estonia, including the long absence of ASFV-positive wild boar, motivated us to conduct the present study. Besides discussing the terminology of (potential) carriers and the impact of such animals on ASF epidemiology, we aimed to understand the cause of the ASFV re-emergence in Estonia. We therefore investigated the probability of ASFV detection, potential ASFV circulation at a low prevalence, and the role of seropositive wild boar in the course of ASF in Estonia. Accordingly, we investigated the three hypotheses:

1. Persistence: ASFV was present in wild boar or the environment throughout the period with no ASFV detections, but at a low prevalence (≤1% or 2%, respectively).
2. Virus carrier-hypothesis: seropositive wild boar could remain infectious and, thus, kept the epidemic going.
3. Reintroduction: the virus was absent for 1.5 years and newly introduced.

## 2. Materials and Methods

### 2.1. Study Areas and Data

**Figure 1:**
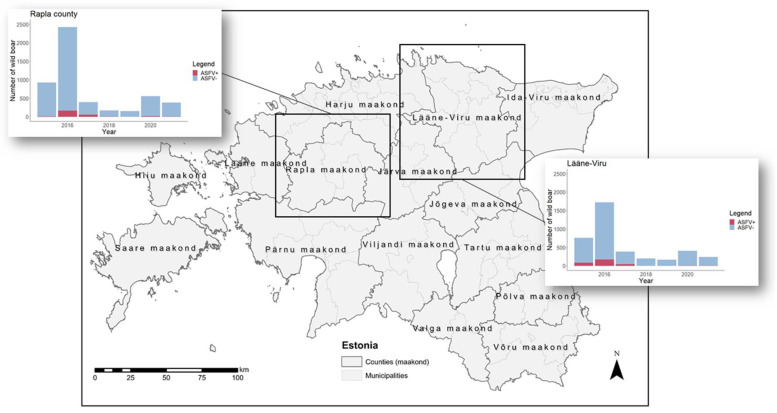
Map of Estonia. The two study areas Rapla County and Lääne-Viru County are highlighted by black rectangles. For each county, the figure shows the numbers of virological investigations from 1 January 2015 through to 30 April 2021. Red bars represent ASF virus positive wild boar by a PCR test, blue bars indicate negative PCR test results.

Estonia consists of 15 counties divided into 79 municipalities (Figure 1). The first study area, Rapla County, is located in central Estonia, and consists of four rural municipalities. The second study area, Lääne-Viru County, is located in northeast Estonia, and consist of eight municipalities. Information on samples taken from hunted wild boar and from passive surveillance (found dead, shot sick, or died in a road traffic accident) was extracted from the CSF/ASF wild boar surveillance database of the European Union (https://surv-wildboar.eu, accessed on 8 December 2021) for all of Estonia, for the period January 2015 to April 2021 (*n* = 56,622). For each data record, information about the origin of the sample (wild boar hunted or found dead), the sampling location, and the results of serological and virological laboratory tests was available. For the detection of ASFV-specific antibodies in hunted wild boar, a commercially available blocking ELISA (Ingezim PPA COMPAC, Ingenasa, Madrid, Spain) was used. If the ELISA result was positive or inconclusive, the sample was retested by an indirect immunoperoxidase technique (IPT) for confirmation using the protocols provided by the European Union Reference Laboratory (EURL) for ASF (CISA-INIA, Valdeolmos, Spain). Real-time PCR was applied to detect the ASFV genome in wild boar hunted or found dead according to the protocols reported by Tignon et al. [26]. Whenever possible, positive PCR results were confirmed by an alternative real-time PCR assay, the Universal Probe Library (UPL) real-time PCR [27]. The samples that finally yielded an inconclusive test result in serological or virological laboratory tests were excluded from the analyses (*n* = 121).

Wild boar population estimates for each county were calculated based on the number of hunted wild boar ($N_{hunt})$. We assumed that a proportion of 45% of the total wild boar population will be hunted or die during one year ($p_{dead}=0.45$) [28]. In addition, we assumed that for 90% of wild boar, the cause of death is hunting ($p_{hunt}=0.9$), whereas 10% of the animals die due to other reasons (age, disease, or predation) [29]. Thus, the wild boar population $N_{pop}\;$ was calculated as

(1) $$N_{pop}=\frac{1}{p_{dead}}\;\cdot \frac{1}{p_{hunt}}\;\cdot N_{hunt}=2.469\;\cdot \;N_{hunt}.$$

Finally, $N_{pop}\;$ was rounded up to the next larger whole number.

### 2.2. Persistence

To evaluate, if ASFV might have been present, undetected at a low prevalence between the last ASFV-positive cases in February 2019 and the new emerging cases in August 2020, we calculated the sensitivity of the surveillance system and the confidence in freedom.

#### 2.2.1. Sensitivity of the Surveillance System

The sensitivity of the surveillance system on a county level was calculated using Epitools for simple risk-based surveillance, in which a high-risk population is targeted [30,31]. To this end, the population was divided into two groups according to their risk of ASF occurrence: (1) The high-risk group included all dead wild boar (carcasses) and (2) the low-risk group contained all living wild boar. Based on the surveillance data, the proportion of wild boar found dead, shot sick due to sickness, or killed in road traffic accident from all wild boar considered for ASF surveillance decreased from 1.7% in 2018 to 0.6% in 2021 (only data until April 2021 were considered). To account for a potential underestimation of the number of dead wild boar (represented by ‘found dead’ included in the surveillance data), we assumed a proportion of dead wild boar (carcasses) of 3% in this study. 

To calculate the surveillance sensitivity, the following information was needed as input:

1. Relative risk. This measure describes the relative risk of ASF-infected wild boar in the high-risk group, relative to the risk of ASF-infected wild boar in the low-risk group. Based on the surveillance data, the risk that wild boar in the high-risk group are ASFV-infected was estimated to be 60 times higher compared to the risk that hunted wild boar were ASFV-positive (low-risk group).
2. Test characteristics. The sensitivity of the test performed on individual wild boar (i.e., of the virological laboratory test) was assumed as 0.999. The specificity of the surveillance system was assumed equal to 1, expecting that all positive wild boar were followed up by a confirmatory test to ensure that they were not false positive.
3. Number of animals tested in the high- and low-risk group. The surveillance sensitivity was calculated for 2018, 2019, and 2020. However, in each year, only those months during which no ASFV-positive wild boar was detected in the two study areas, were included in the calculations. The number of animals tested in the high- and low-risk group was, thus, calculated for the considered time periods, based on the surveillance data.

As an output, the surveillance sensitivity was recorded as the probability that the surveillance system will detect at least one infected wild boar, if ASF is present at design prevalence values of 1% or 2%, respectively. The design prevalence of 1% or 2% was chosen, because these values correspond to an effective probability of infection (EPI) in the high-risk group (i.e., in wild boar carcasses) of 21.7% or 43.3%, respectively, and an EPI in the low-risk group (i.e., in hunted wild boar) of 0.4% or 0.7%. A surveillance sensitivity above 95% was considered sufficient to detect ASFV in the wild boar population.

#### 2.2.2. Confidence in Freedom from ASF

In addition, the confidence in freedom from infection with ASFV for multiple periods was estimated using Epitools [30,32]. Using the estimated surveillance sensitivity for the three considered periods, we evaluated the ability of the surveillance system (confidence in freedom) to detect ASFV at the two design prevalence values of 1% and 2%. The initial prior confidence in freedom was set to 0.5, since no information was available. 

No information was available regarding the probability of ASFV introduction into the wild boar population. Assuming that a new introduction of ASFV into two non-neighboring counties could be considered as independent, we estimated the probability of introduction for Estonia as a whole $PI_{EE}\;$ as:

(2) $$PI_{EE}\geq 1-{\left(1-PI_{county}\right)}^{n},$$

with $PI_{county}\;$ as the assumed probability of introduction per county and *n* as number of counties in Estonia (*n* = 15). Conservative values for $PI_{EE}\geq 79\%$ and $PI_{county}=10\%\;$ were used and complemented by a sensitivity analysis to assess the uncertainty when choosing the above values.

A confidence in freedom from ASFV above 95% was considered sufficient.

### 2.3. Virus Carrier Hypothesis

To evaluate the potential role of ASF-seropositive wild boar in maintaining the epidemic, we assumed that ASF-seropositive wild boar might still be intermittent infectious. Thus, the hypothesis is that the number of ASFV-positive wild boar increases during the subsequent period, once a seropositive wild boar has been detected (positive index case). We therefore examined if the number of investigated wild boar was sufficient to detect ASFV-positive wild boar, once an index case had occurred, by calculating the detection probability. Furthermore, a cluster analysis was performed to identify potential spatial and spatiotemporal clusters of seropositive wild boar.

#### 2.3.1. Detection Probability

We estimated the probability of detecting at least one ASFV-positive wild boar after a seropositive wild boar had been hunted (positive index case) and compared the detection probability to the estimates obtained after hunting a seronegative wild boar (negative index case). Therefore, for every hunted wild boar that was investigated serologically during the study period (index wild boar), the number of virological investigations was recorded for periods of 90 and 120 days after the date of the serological investigation of the index wild boar. The result was used to estimate the detection probability for each index wild boar, i.e., the sensitivity of detecting ASFV in the two considered periods. Again, we used design prevalence values of 1% or 2%, respectively. The calculation was based on a previously presented formula for surveys to substantiate freedom from disease considering imperfect test characteristics and a finite population size [33]. The formula requires the wild boar population (estimated as detailed above), the number of virological investigations in the considered time period (calculated based on the surveillance data), the expected number of ASFV-positive wild boar in the population (equals the design prevalence multiplied with the population size $N_{pop}$) and the sensitivity of the performed virological laboratory test (sensitivity = 0.999) as input parameters. Results were presented as histograms of the resulting detection probabilities. Values above 95% were considered as sufficient to detect ASFV-positive wild boar at the chosen design prevalence value.

#### 2.3.2. Cluster Analysis

To investigate if more seropositive wild boar were found in the two Estonian study areas compared to other Estonian counties, a cluster analysis was performed. To test for global clustering, we used the Tango test [34] and Oden’s I pop, which account for varying wild boar population densities in Estonia [35]. SaTScan analysis was used to identify spatial, temporal, and spatiotemporal clusters in serological positive wild boar [36].

### 2.4. Software

**Figure 2:**
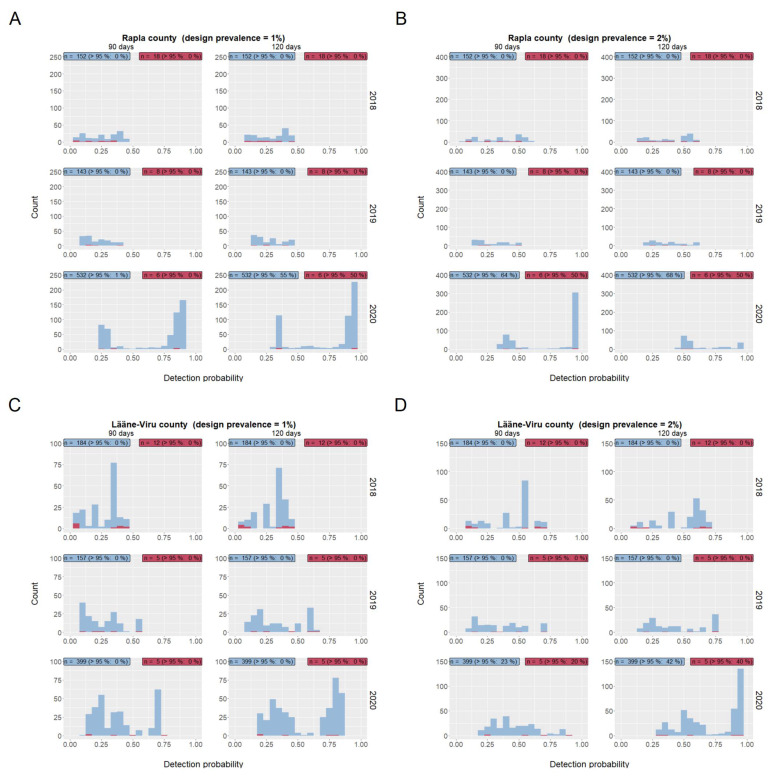
Map of Estonia. The two study areas—Rapla County ((**A**): design prevalence 1% and (**B**): design prevalence 2%) and Lääne-Viru County ((**C**): design prevalence 1% and (**D**): design prevalence 2%)—are highlighted by black rectangles. For each county, the figure shows the numbers of virological investigations from 1 January 2015 through to 30 April 2021. Red bars represent ASF virus positive wild boar by a PCR test, blue bars indicate negative PCR test results.

**Figure 3:**
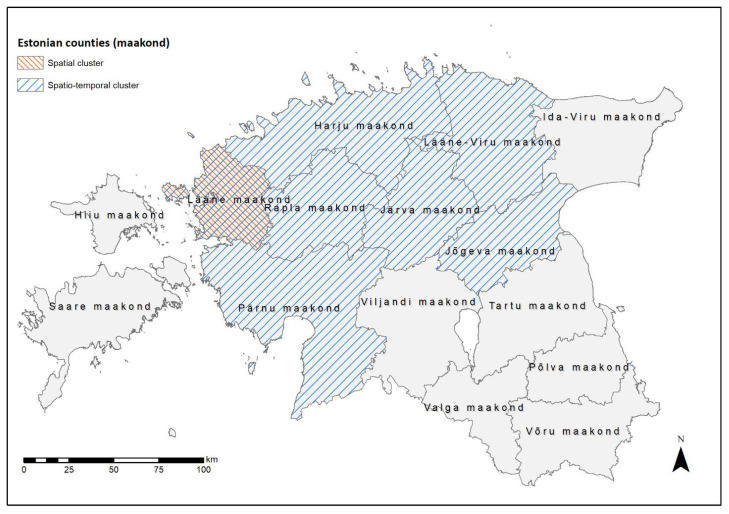
Significant spatial (red) and spatiotemporal (blue) clusters of seropositive wild boar detected by SaTScan analysis in Estonia.

If no specific software was mentioned in the previous sections, statistical analysis was performed using the software R version 4.0.3 (http://www.r-project.org, accessed on 9 January 2021), which was also used to generate figures. The following R packages were used: lubridate [37], RSurveillance [38], ggplot2 [39], gridExtra [40], ggpur [41], spdep [42], and DCluster [43]. Figure 1, Figure 2 and Figure 3 were generated using ArcGIS ArcMap 10.8.1 (ESRI, Redlands, CA, USA, http://www.esri.com/, accessed on 9 January 2021).

## 3. Results

The sensitivity of the surveillance system and the confidence in freedom from ASF were investigated to assess, if ASFV might have gone undetected at a low prevalence between the last ASFV-positive cases in February 2019 and the cases that re-emerged since August 2020.

### 3.1. Persistence

#### 3.1.1. Sensitivity of the Surveillance System

In both study regions, the number of wild boar carcasses investigated for ASFV in each considered period varied between zero and four (Table 1). The surveillance sensitivity for an assumed design prevalence of 1% was below 95% in both study areas for all three considered periods (Table 1). Assuming a design prevalence of 2%, in Lääne-Viru County, the estimate of the surveillance sensitivity remained above 95% in two periods, and was thus considered sufficient to detect ASFV circulation.

#### 3.1.2. Confidence in Freedom from ASF

For an assumed design prevalence of 1%, the estimated confidence in freedom considering the three periods individually was below 90% in both study areas (Table 2). Increasing the design prevalence to 2% led to sufficient levels of confidence (i.e., above 95%) in Lääne-Viru for two considered time periods (2018 and January–November 2020, Table 2).

Considering multiple consecutive time periods, we achieved a cumulative confidence in freedom after three periods for both design prevalence values in Lääne-Viru County (Table 2). In contrast, in Rapla County, a cumulative confidence in freedom above 95% was only observed for 2% design prevalence (Table 2).

As no information on the probability of introduction was available, a sensitivity analysis was performed. Choosing lower values for the probability of introduction (e.g., $PI_{county}=$5% ($PI_{EE}\;\geq 54\%)$) led to increased values for the confidence in freedom. Increasing the introduction probability (e.g., $PI_{county}=15\%$ ($PI_{EE}\geq 91\%)$) led to decreasing values for the confidence in freedom. However, compared to the probability of introduction of $PI_{county}=10\%$, the result regarding confidence in freedom altered only marginally.

### 3.2. Virus-Carrier-Hypothesis

#### 3.2.1. Detection Probability

In Rapla County, the number of serologically tested wild boar varied between 151 in 2019 and 538 in 2020 (all of the tested wild boar tested negative for ASFV) (Figure 2A,B). Between 6 and 18 animals were found seropositive. These animals were considered as potential ASFV-carriers. In Rapla County, the estimated detection probability was below 95% in 2018 and in 2019, regardless of the chosen design prevalence value of 1% or 2%. In 2020, a detection probability above 95% was reached for 3 of 6 index wild boar (50%), assuming a design prevalence of 1% and considering a period of 120 days after the detection of the index case (Figure 2A). Assuming a design prevalence of 2% led to detection probabilities above 95% also for a period of 90 days after the detection of the index case in 2020 (Figure 2B).

In Lääne-Viru County, between 162 and 404 wild boar were tested, and 5 and 12 were serologically positive, but ASFV-negative (Figure 2C,D). In none of the scenarios described above for Rapla County, a detection probability above 95% was reached in Lääne-Viru County, assuming a design prevalence of 1% (Figure 2,C). Increasing the design prevalence to 2% led to a proportion of index wild boar with detection probabilities above zero in 2020 (Figure 2,D). In both study areas, we did not observe a difference in the proportion of index wild boar leading to detection probabilities above 95%, following a seropositive or a seronegative index wild boar (Figure 2).

In general, the median proportion of cases on Estonian county level, in which the detection probability was above 95%, was zero, except for one situation (Table 3): in 2020, the proportions of cases, in which the detection probability was above 95%, ranged between 0 and 83% on the county level in Estonia, assuming a design prevalence of 2%. A median of 42% was calculated regardless of the virological status of the index wild boar.

#### 3.2.2. Cluster Analysis

Both global cluster tests, the Tango test and Oden’s I pop showed *p*-values below 0.05 (Tango test *p* = 0.010, Oden’s I pop *p* < 0.001). Thus, both tests indicated that statistically significant clusters of seropositive wild boar existed in Estonia. A spatial cluster covering Lääne County (not to be confused with Lääne-Viru County) with a statistically significantly higher number of seropositive wild boar was identified by SaTScan analysis (Figure 3). Lääne County is located in direct neighborhood to the study area Rapla County (Figure 1). The temporal cluster analysis found the whole of Estonia as a cluster in March 2019. Combining spatial and temporal analysis led to a cluster for February–March 2019 covering seven counties (Figure 3). Thus, no link could be identified to the periods, when the new ASFV cases had occurred.

## 4. Discussion

After five years of ASFV circulation and a decreasing ASFV prevalence in Estonia, no more ASFV-positive wild boar were detected from March 2019 until August 2020. During that time, seropositive wild boar were still hunted, although at a low prevalence [17]. Newly detected wild boar carcasses that had tested positive for ASFV were found in two counties, in which the virus had not been detected for 28 months in Rapla County and 37 months in Lääne-Viru county. Thus, the hope that freedom from ASF in wild boar might be a realistic task was destroyed. The question was raised—how did ASFV re-emerge in Estonia? We set up the following hypotheses: (1) Persistence: ASFV was present all the time in wild boar or in the environment (e.g., carcass), but at a low prevalence. (2)Virus carrier-hypothesis: seropositive wild boar may be infectious and can maintain the epidemic. (3) Reintroduction: ASFV was not found in the entire country for 1.5 years and then newly introduced.

To evaluate the probability of ASFV detection, the sensitivity of the surveillance system and the confidence in freedom from ASF were calculated. Several assumptions were made in these calculations. The chance to detect ASFV in dead wild boar was estimated to be 60 times higher than in apparently healthy animals. This was estimated based on the Estonian surveillance data. Similarly, a significant higher probability to detect ASFV in wild boar carcasses than in animals shot apparently healthy was found in several other studies [7,12,44,45]. Moreover, the assumed proportion of wild boar carcasses (3%) relative to all sampled wild boar was defined based on available surveillance data, and is similar to the data composition in other countries [16,46,47]. In a recent analysis by the European Food Safety Authority [48], this proportion was estimated to be only 1%. The higher proportion in our study may at least in part be explained by the fact that our estimates include the assumed proportion of undetected wild boar carcasses. Furthermore, following the World Organisation for Animal Health [49], almost perfect test sensitivity and specificity were assumed for calculating the sensitivity of the surveillance system. The chosen values were supported by findings by Schoder et al. [50]. To calculate the confidence in freedom, the estimated probability of introduction was incorporated. Since substantiated data are lacking, we chose a rather conservative value of 10% per county. To account for the uncertainty of the chosen values, the influence of this choice on the results was tested by performing a sensitivity analysis. This analysis investigated introduction probabilities for the whole of Estonia between 54% and 91%. Larger values were regarded as unrealistic. By using the lower value of 54%, we attempted to reflect the current ASF situation in neighboring countries and in Estonia as a whole. If the probability of introduction was even lower than anticipated, the confidence in freedom would increase and thus lead to the same conclusion.

Only in Lääne-Viru County, but not even there in all three periods, the sensitivity of the surveillance system was high enough to detect the disease at a design prevalence of 2%. It must therefore be assumed that the surveillance system was not sufficient to detect virus circulation at this low prevalence. When we took previous periods into account when calculating the confidence in freedom from ASFV, the results indicated that the circulating virus should have been detected over time, if a prevalence of 2% was assumed. The result for a prevalence of 1%, in which ASFV circulation is likely to be missed by the surveillance system, corroborates the analyses of the European Food Safety Authority [48]. In several studies, it was found that the wild boar population density significantly decreases during an ASF epidemic [15,22,44,46]. The low ASFV prevalence combined with a low population density may suggest that the epidemic is fading. However, considering the calculations for confidence in freedom, ASFV may be re-discovered after some time, if the prevalence starts to rise and exceeds the detection threshold [48]. Thus, it seems possible that the virus circulation at some stage exceeded the detection limit in the two counties under study again, so that the virus was detected. However, it could be expected that the number of detected carcasses would further increase in case of exceeding the detection threshold.

Moreover, it seems unlikely that ASFV circulated throughout Estonia for more than 1 year at such a low level and remained undetected, particularly when considering the low wild boar population density and assuming the wild boar habitat infection cycle [10].

The role of seropositive wild boar and its potential role as a “carrier” in the course of ASF has been controversially discussed for several years [24]. Blome et al. [5] provided clear definitions, calling a “true” survivor an animal, in which ASFV-specific antibodies, but no ASFV is detected. The probability that such animals shed an infectious virus and play a role as virus-carriers in disease spread is virtually zero. Eble et al. [21] argued, however, that animals that survive an ASFV infection (i.e., showing ASFV-specific antibodies), could shed ASFV and, thus, transmit the disease. Therefore, they called these animals ASFV-carriers. The pigs in their study had tested positive for ASFV during the whole study period, which was only 55 days. It therefore remains open for how long virus shedding might have continued. Thus, the findings of Eble et al. [21] do not contradict the statements by Blome et al. [5], who argued that ASFV or viral genome can be detected for approximately 60- and 100-days post-infection, respectively, and that an infected wild boar could play a role as virus-carrier during that period. Consequently, if a virus-carrier in the context of ASF is an animal that survived an ASFV-infection, but still carries and sheds detectable amounts of ASFV for a certain period, then there is no doubt that virus-carrier animals exist. If, however, all wild boar that have survived ASF (as shown by the presence of ASFV-specific antibodies in apparently healthy animals) are regarded as potential carriers, even in absence of detectable virus, more evidence is needed. Gallardo et al. [51] hypothesized that ASFV might be reactivated under immunosuppression, stress, or death and, thus, these potential carriers might play a role in ASF persistence in swine-populations. So far, evidence for ASFV transmission has neither been obtained in animals surviving more than 100 days, nor has excretion of an infectious virus been detected from such animals [20,23]. It cannot be excluded, however, that a very small number of wild boar might transmit ASFV even after 100 days. Moreover, sample matrix and quality may have influenced the probability of detecting low amounts of viral genomes or viral genomes confined to one organ or organ system. In this respect, screening of various tissues could aid detection of potential virus-carriers.

In this study, we tested the role of potential ASFV-carriers in Estonia (i.e., wild boar with ASFV-specific antibodies, but not ASFV-positive) by estimating the detection probability of ASFV-positive wild boar emerging after the occurrence of a seropositive wild boar. Since there is no doubt that surviving animals can transmit ASFV for some time, we only evaluated the hypothesis that seropositive, but ASFV genome negative wild boar may spread ASF and, therefore, represent apparently healthy ASFV-carriers. We assumed that ASFV-positive animals would be detected in a period of 90 or 120 days after detection of the seropositive wild boar, if these animals were infectious. These time spans were chosen because ASFV has been detected 60–70 days post-infection and viral genome up to approximately 100 days post-infection [20,23]. These calculations were conducted only for the time periods in which no ASFV-positive wild boar were detected in Estonia (between February 2019 and July 2020). The significantly decreased population density was considered. The probability to detect ASFV-positive wild boar after a seropositive animal had been found was too low to exclude the possibility that seropositive animals could spread ASF.

To further investigate possible causes for the re-emergence of ASFV in the two affected counties with regard to seropositive animals and their potential to spread ASFV, cluster analyses were performed. In the spatial analysis, a statistically significant cluster was identified in a county bordering Rapla County. In this county (Lääne County), the last ASFV-positive wild boar was detected in February 2019, representing the last ASFV-positive animal in Estonia for more than 1 year. An increasing seroprevalence subsequent to a decreasing ASFV prevalence was described in several countries [16,22,46]. These findings may explain the identified cluster in Lääne County, which could simply be a result of the temporal course of the epidemic on a county level, without any epidemiological link to the new occurrence of ASFV in Rapla County. The distance between the last ASFV-positive wild boar in February 2019 and the first one detected in August 2020 was approximately 75 km. The European Food Safety Authority [48] found a median speed of spread of 3–12 km/year in the affected Member States. In Poland, ASF has also been described as spreading rather slowly [52,53]. These findings, the generally limited home range of wild boar [54,55], and their tendency to avoid contact between individual packs [56], support the hypothesis that the re-emergence of ASFV in Rapla County may be independent from the cases in Lääne County in February 2019. The only significant cluster obtained from the spatio-temporal cluster analysis was identified at the beginning of the study period, in which Estonia was potentially free from ASFV and included almost the entire country. Similar to the spatial and temporal cluster analysis, but on country level, this result is likely to be due to the temporal course of ASF, i.e., the decrease of the ASFV prevalence and the subsequent increase in the proportion of seropositive animals [16,22]. Thus, the spatiotemporal expansion is not surprising. The lack of further spatiotemporal clusters at a later stage suggests the absence of ASFV circulation and makes it unlikely that seropositive animals played a role in virus transmission. In addition, clusters could also be the result of different ASFV strains with varying survival rates. Thus, a less virulent strain might lead to a higher number of seropositive wild boar and consequently results of cluster analysis should be interpreted with care.

Our results suggest that it was unlikely that ASFV circulated at a low prevalence without detection for 1.5 years (Persistence). Unfortunately, the study did not yield unambiguous results regarding the potential role of seropositive wild boar in transmitting ASFV (Virus carrier-hypothesis). Although it seems unlikely that seropositive, but ASFV negative wild boar shed ASFV and spread the disease, this could not be completely ruled out. It still needs clarification if (1) a considerable proportion of infected wild boar can survive ASF, still harboring and shedding the virus for longer periods than currently discovered, and thus playing a significant role in the further spread of ASF. Furthermore, the question if (2) ASFV could be reactivated in seropositive wild boar under immunosuppression, stress or in case of death [57] has to be pursued further. To find scientific evidence regarding these questions, it will be inevitable to conduct long-term experimental studies.

The third hypothesis (Reintroduction of ASFV) seems the most likely explanation for the re-emergence of ASF in Estonia. The virus could have been newly released locally e.g., by dropping infected wild boar meat from illegally hunted wild boar. Moreover, the virus could have been introduced from neighboring countries, where ASF is still present in various regions [58]. The outbreak in Lääne-Viru County was detected several months after the Rapla County outbreak and could be the result of human mediated transmission from there. Preliminary results of molecular sequencing of ASFV strains emerging in Rapla and Lääne-Viru County in 2020 indicate a 100% match to the Georgia 2007/1 strain [59]. This strain currently circulates in Russia, Belarus, Poland, and several other countries. Thus, reintroduction of this virus seems possible. Yet, the genome of ASFV has proven to be very stable, so that available sequencing results do not allow rejecting the other hypotheses.

Despite the huge effort to test various hypotheses that might explain the re-emergence of ASF in Estonia, we must confess—with Socrates—that we (only) know that we know nothing. Yet, appropriate consequences have to be drawn to improve ASF control in Estonia. Collaborative and interdisciplinary efforts are needed more than ever to develop and investigate further hypotheses to increase the chances of combating ASF successfully. With increasing availability of ASFV full-length sequences [60], further molecular epidemiology can help to solve open questions about potential virus origins. Nevertheless, the epidemiological course of the disease in affected countries must be analyzed and jointly discussed with scientists to increase the knowledge and improve our understanding of ASF and its epidemiology.

## Figures and Tables

**Table 1:** Number of investigated wild boar and sensitivity of the surveillance system (risk-based) assuming design prevalence (dp) values of 1% and 2% in Rapla County and Lääne-Viru County for the considered periods. Values above 95% are marked in bold.

Study Area	Time Period	Number of Wild Boar Investigated for ASFV		Surveillance Sensitivity (in %)	
		Hunted	Found Dead	dp = 1%	dp = 2%
Rapla County	March 2018–December 2018	81	1	41.5	68.4
	2019	150	3	72.0	93.8
	January 2020–July 2020	171	0	46.1	71.0
Lääne-Viru County	2018	196	4	81.4	**97.5**
	2019	163	1	56.5	82.6
	January 2020–November 2020	313	4	87.8	**98.9**

**Table 2:** Cumulative confidence in freedom assuming a probability of introduction of 10% and design prevalence (dp) values of 1% and 2% in Rapla County and Lääne-Viru County for the considered periods. Values above 95% are marked in bold.

Study Area	Individual Time Periods	Confidence in Freedom for Individual Time Periods (in %)		Consecutive Time Period	Cumulative Confidence in Freedom Over Consecutive Periods (in %)	
		dp = 1%	dp = 2%		dp = 1%	dp = 2%
Rapla County	March–December 2018	63.1	76.0	March–December 2018	58.3	72.1
	2019	78.1	94.2	March 2018–December 2019	79.8	**96.8**
	January–July 2020	65.0	77.5	March 2018–July 2020	82.5	**95.9**
Lääne-Viru County	2018	84.3	**97.6**	January–December 2018	81.5	**97.0**
	2019	69.7	85.2	January 2018–December 2019	86.3	**97.5**
	January–November 2020	89.2	**98.9**	January 2018–November 2020	**96.6**	**99.9**

**Table 3:** Median (minimum–maximum) number of serological investigations in Estonian counties that were used to identify index wild boar cases to estimate the probability of detecting ASFV-positive wild boar 90 and 120 days after detection of the index wild boar case. Sero+ indicates estimates after seropositive, but ASFV-negative index wild boar. Sero +/− indicates estimates for the median [minimum-maximum] number of index wild boar cases regardless of the serological test result for ASFV-negative wild boar. For the two periods of 90 and 120 days after detection of the respective index case, the median (minimum–maximum) proportion of the detection probability above 95% is presented for assumed design prevalence (dp) values of 1% and 2%.

	Time Period	Number of Index Wild Boar (Median (Minimum-Maximum))	Days after Serological Investigation of Index Wild Boar	Proportion of Detection Probabilities above 95%	
				dp = 1%	dp = 2%
Sero+	2018	10 (0–71)	90	0 (0–21)	0 (0–45)
			120	0 (0–27)	0 (0–54)
	2019	4 (0–12)	90	0 (0–0)	0 (0–60)
			120	0 (0–50)	0 (0–60)
	2020	4 (0–17)	90	0 (0–35)	0 (0–88)
			120	0 (0–88)	0 (0–88)
Sero+/−	2018	256 (101–1031)	90	0 (0–230)	0 (0–54)
			120	0 (0–27)	0 (0–65)
	2019	244 (83–955)	90	0 (0–0)	0 (0–53)
			120	0 (0–28)	0 (0–64)
	2020	352 (137–1445)	90	0 (0–39)	0 (0–75)
			120	0 (0–67)	42 (0–83)

## Data Availability

The original data used for the analyses can be obtained from the authors after approval by the responsible institutions in Estonia.
